# A reappraisal of *APOE* genetic effects on Alzheimer’s disease risk in the Japanese population: a meta-analysis

**DOI:** 10.1186/s13024-026-00963-z

**Published:** 2026-06-24

**Authors:** Akinori Miyashita, Norikazu Hara, Ai Obinata, Tamao Tsukie, Mai Hasegawa, Takanobu Ishiguro, Kensaku Kasuga, Masataka Kikuchi, Tadafumi Hashimoto, Takahisa Kanekiyo, Takeshi Ikeuchi

**Affiliations:** 1 Department of Molecular Genetics, Brain Research Institute, Niigata University, 1-757 Asahimachi, Chuo-ku, Niigata, 951-8585 Japan; 2 Department of Neurology, Brain Research Institute, Niigata University, Niigata, Japan; 3 Department of Diagnostic Innovation Science, Center for Development of Advanced Medicine for Dementia, National Center for Geriatrics and Gerontology, Obu, Japan; 4 Department of Degenerative Neurological Diseases, National Institute of Neuroscience, National Center of Neurology and Psychiatry, Kodaira, Japan; 5 Department of Neuroscience, Mayo Clinic, Jacksonville, FL USA

## Abstract

Our meta-analysis demonstrates that the risk effect of APOE-e4*4 relative to APOE-e3*3 for Alzheimer’s disease in the Japanese population is approximately 12–15-fold, comparable to that reported in Caucasian populations, rather than greater than 20-fold as previously reported.

**Supplementary Information:**

The online version contains supplementary material available at 10.1186/s13024-026-00963-z.

**To the editor**

The apolipoprotein E (*APOE*) *e4* allele is widely recognized as the strongest genetic risk factor for Alzheimer’s disease (AD), with *e4* homozygosity (e4*4) conferring a substantially increased risk relative to the e3*3 genotype [1]. In Japanese populations, early studies have reported particularly high risk estimates, with odds ratios (ORs) of 33.1 (95% confidence interval [CI], 13.6–80.5) [2] and 21.8 (95% CI, 8.6–55.3) [3] for e4*4 vs. e3*3. However, these estimates were derived from a limited body of evidence: one study [2] was based on a meta-analysis of six reports published between 1993 and 1995 [4–9], while the other [3] recalculated effect sizes based on a subset of studies [4, 5, 7, 8] that partially overlapped with this earlier dataset [2]. This dependence on early and non-independent data sources, combined with relatively small sample sizes, may have contributed to inflated effect estimates. Given the importance of accurate and population-specific risk quantification, a systematic reassessment using aggregated evidence is warranted. In this study, we conducted a meta-analysis of published case-control studies in Japanese populations to more precisely estimate the effect size of e4*4 relative to the e3*3 genotype.

Studies were identified through systematic searches of PubMed and Web of Science (WOS) in accordance with PRISMA guidelines [10]. Details of the meta-analysis protocol are available via the Open Science Framework (https://doi.org/10.17605/OSF.IO/U37QF). The processes of literature search and study selection are illustrated in Supplementary Fig. 1, and the overlap of studies with PubMed identifiers (PMIDs) identified from PubMed and WOS is summarized in Supplementary Table 1. After excluding 14 non-English articles, all remaining 242 English-language articles were obtained and assessed by full-text review. Eligible studies were selected based on predefined criteria. Contingency table data for *APOE* genotypes were extracted and cross-checked; when not directly available, counts were derived from reported genotype frequencies. For classification into early-onset AD (EOAD) and late-onset AD (LOAD), we adopted the definitions provided in each original study when available; if such classification was not specified, the data were treated as AD and corresponding contingency tables were constructed. The contingency tables of the final 21 included studies (Supplementary Table 2, Supplementary References) were cross-checked to ensure data accuracy.

Among the 242 English-language articles, two studies using subjects derived from the Hyogo Institute for Aging Brain and Cognitive Disorders (Himeji, Japan) were identified (PMIDs: 10329743 and 16233903). As substantial overlap of subjects between these studies was strongly suspected, we included only the more recent study (published in 2006; PMID: 16233903) for the meta-analysis, excluding the earlier report (published in 1999; PMID: 10329743). Multiple studies have been conducted using cohorts from the Japanese Genetic Study Consortium for Alzheimer’s Disease (JGSCAD), and the National Center for Geriatrics and Gerontology (NCGG). To minimize potential bias arising from overlapping use of subjects across these studies, we limited our analysis to two representative, recent, and large-scale reports by Asanomi et al. (2019) [11] and Miyashita et al. (2025) [12]. In the study by Asanomi et al. [11]., *APOE* genotype contingency tables were presented separately for the NCGG (Table S2) and JGSCAD (Table S4) cohorts, which allowed a meta-analysis combining these two cohorts (Supplemental Table 6 in [12]). The resulting pooled ORs with 95% CIs were used for reference and comparison with the EOAD, LOAD, and AD results in the present study (Fig. 1B, Supplementary Table 3). The study by Miyashita et al. [12]. also included NCGG and JGSCAD subjects, encompassing those used in Asanomi et al. [11]., but provided only aggregated *APOE* genotype data for case (AD) and control groups without cohort-specific stratification. Accordingly, only crude ORs with corresponding 95% CIs could be calculated from this study. The study by Miyashita et al. included substantially larger sample sizes (cases: 6,261; controls: 16,331) [12] compared with Asanomi et al. (cases: 4,146; controls: 3,267) [11]. To avoid subject overlap and minimize the disproportionate influence of these markedly larger studies, both were excluded from the primary meta-analysis of the EOAD, LOAD, and AD groups and retained solely for reference and comparison (Fig. 1B, Supplementary Table 3).

**Fig. 1:**
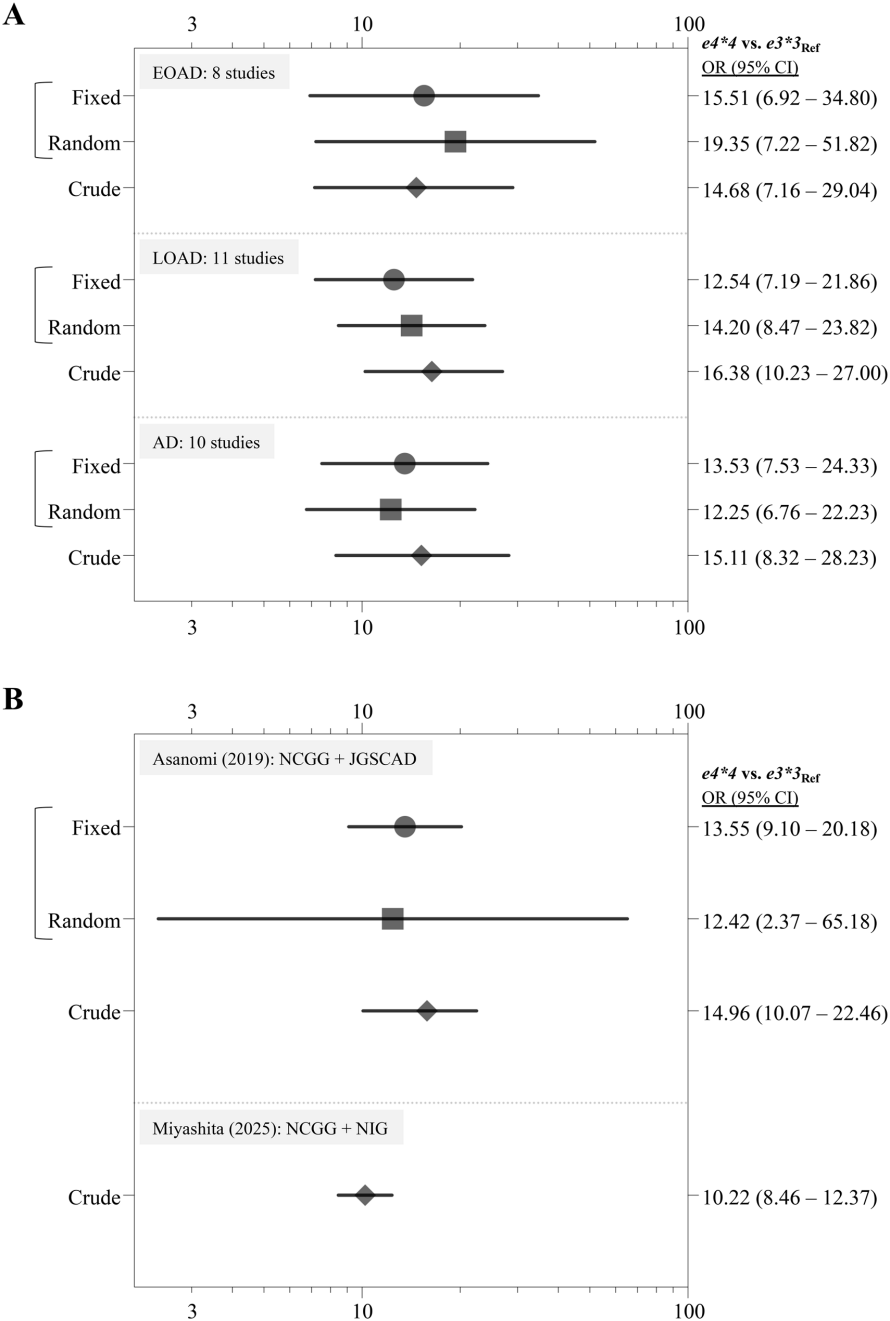
Meta-analysis of APOE-e4*4 vs. APOE-e3*3 in Japanese AD. (**A**) Forest plots showing ORs with 95% CIs for e4*4 vs. e3*3 (reference) in EOAD, LOAD, and AD, estimated using fixed-effects and random-effects meta-analysis models. Crude ORs with corresponding 95% CIs (Supplementary Table 3) are also shown for reference in each forest plot. (**B**) For comparison with (**A**), ORs with 95% CIs from two recent large-scale Japanese datasets [11, 12] are presented separately. As described in Supplementary Fig. 1, these datasets were excluded from the primary meta-analysis in (**A**) because their substantially larger sample sizes relative to earlier reports could introduce bias. For the dataset by Asanomi et al. [11]., results from fixed- and random-effects meta-analyses, as well as a crude OR, are presented. For the dataset by Miyashita et al. [12]., only the crude OR is shown, as individual-level APOE genotype data were not available [12], precluding meta-analysis. The forest plots for the Asanomi dataset (2019) and the Miyashita dataset (2025) were constructed using numerical data reported in Supplementary Table 6 and Table 3 of Miyashita et al. [12]., respectively

Pooled ORs and 95% CIs were estimated using StatsDirect software (v.4.0.4) under fixed- and random-effects models. Heterogeneity was assessed using Cochran’s Q test and the I² statistic, and potential publication bias was evaluated by funnel plot inspection and by the Begg-Mazumdar, Egger’s, and Harbord-Egger tests (Supplementary Fig. 3, Supplementary Table 4). Crude ORs with 95% CIs were also calculated using GraphPad Prism (version 11.0.0).

As summarized in Supplementary Tables 2 and 3, the 21 studies included in our analysis comprised several hundred cases and several thousand controls, with the e4*4 genotype being rare among controls but more frequent among cases. The pooled ORs under the fixed-effects model were 15.51 (95% CI, 6.92–34.80) for EOAD, 12.54 (95% CI, 7.19–21.86) for LOAD, and 13.53 (95% CI, 7.53–24.33) for AD, with similar estimates obtained under the random-effects model (Fig. 1A, Supplementary Table 4). These results indicate that *e4* homozygosity confers a roughly 12- to 15-fold increase in risk, which is still substantial but markedly lower than the much higher ORs of 33.1 [2] and 21.8 [3] reported in early meta-analyses. Figure 1B presents results from two large contemporary cohorts for comparison with our findings: Asanomi et al. reported pooled ORs of 13.55 (95% CI, 9.10–20.18) under a fixed-effects model and 12.42 (95% CI, 2.37–65.18) under a random-effects model, as well as a crude OR of 14.96 (95% CI, 10.07–22.46) [11], and Miyashita et al., for whom only crude estimates were available, reported an OR of 10.22 (95% CI, 8.46–12.37) [12] (Supplementary Table 3). These independent estimates closely match our meta-analysis results and support an approximately 12- to 15-fold effect size. Visual inspection of funnel plots did not reveal clear evidence of asymmetry (Supplementary Fig. 3), and statistical tests showed no significant evidence of publication bias (Supplementary Table 4).

Risk estimates for additional genotype, carrier, and allele-based comparisons are also provided in Supplementary Tables 3 and 4. These analyses include *e4* carriers and the protective effects of the *e2* and *e3* alleles, but do not materially alter the overall conclusion that the e4*4 genotype confers the greatest risk of AD among the *APOE* categories examined. Nevertheless, the results for *e4* heterozygosity warrant brief consideration because they are frequently cited in the literature and yielded higher effect estimates in Japanese LOAD than in major studies of European ancestry populations. For *e4* heterozygosity (e3*4 vs. e3*3), the pooled ORs in Japanese LOAD were 5.11 under the fixed-effects model and 5.13 under the random-effects model (Supplementary Table 4), slightly higher than those reported for individuals of European ancestry (3.2 [2], 4.3 [3], and 3.46 [13]). However, this difference should be interpreted cautiously, as variations in study design, diagnostic criteria, and control age distributions may influence OR estimates. Moreover, the moderate heterogeneity among Japanese LOAD studies (I² = 40.0%; Supplementary Table 4) suggests that methodological and demographic differences across cohorts may have contributed to the relatively elevated pooled OR estimates.

While the e4*4 genotype remains the strongest *APOE*-associated risk factor for AD, its effect is less extreme than previously reported [2, 3], providing a more realistic risk estimate for Japanese populations. Importantly, these effect sizes are comparable to those in Caucasian populations [2, 3, 13] but differ from those observed in African American and Hispanic populations [2, 13]. Similar effect sizes have been reported in Chinese cohorts. Liu et al. [14]. conducted a meta-analysis involving 20 studies and reported an OR of 11.71 (95% CI, 6.38–21.47). By contrast, the Korean estimate reported by Choi et al. [15]. was based on a single large cohort (*n* = 17,433) and yielded an OR of 26.66 (95% CI, 18.4–38.7) for *e4* homozygosity, substantially higher than the estimates observed in Japanese (this study) and Chinese [14] populations. A formal meta-analysis of Korean studies is warranted to obtain a more reliable estimate of the effect size in this population.

In conclusion, our meta-analysis indicates that *e4* homozygosity confers an approximately 12- to 15-fold increased risk of AD in Japanese populations, substantially lower than previous estimates exceeding 20-fold [2, 3]. Together with findings from Chinese cohorts [14], these results suggest that the effect of *e4* homozygosity may be broadly similar across East Asian populations, although further evidence from Korean populations is needed to clarify potential population-specific differences.

## Supplementary Information

Below is the link to the electronic supplementary material.

Supplementary Material 1: Supplementary Figure 1. Flow diagram of study selection and overlap between PubMed and WOS. Flow diagram illustrating study identification, screening, and eligibility assessment for the meta-analysis of *APOE* genotype effects in Japanese AD. Literature searches were conducted using PubMed and the WOS Core Collection with the query strings shown. A total of 241 records were identified from PubMed and 213 from WOS, and the overlap among records with PMIDs is indicated. After exclusion of non-English articles (n = 14) and studies lacking sufficient APOE genotype data in both case and control groups (n = 200), and after resolving overlapping cohorts (NCGG/JGSCAD and HI-ABCD), 21 eligible studies were finally included in the primary meta-analysis (EOAD, n = 8; LOAD, n = 11; AD without age stratification, n = 10). Full bibliographic details of the 21 included studies are provided in Supplementary References and correspond to the order of the “Literature ID” entries in Supplementary Table 2. Funnel plots for the e4*4 vs. e3*3 comparisons are shown in Supplementary Figure 3, and publication bias statistics for all comparisons are provided in Supplementary Table 4. The two most recent large-scale Japanese studies involving NCGG/JGSCAD subjects were excluded from the primary meta-analysis (Fig. 1A) and retained for reference only, as their substantially larger sample sizes relative to earlier reports could disproportionately influence pooled estimates (Fig. 1B).
Supplementary Figure 2. Forest plots of study-specific and pooled ORs (95% CIs) for APOE-e4*4 vs. APOE-e3*3 in Japanese AD. Forest plots showing study-specific ORs with 95% CIs for e4*4 vs. e3*3 (reference) in Japanese EOAD (A, B), LOAD (C, D), and AD (E, F). Fixed-effects model results are prensented in (A), (C), and (E), and random-effects model results in (B), (D), and (F). Squares indicate study-specific point estimates (with size proportional to the study weight in the corresponding meta-analysis model), horizontal lines represent 95% CIs, and diamonds indicate pooled ORs estimated from meta-analysis. Each study is labeled by a Literature ID; details are provided in Supplementary Table 2. Studies marked as “excluded” in the figure were omitted from the corresponding meta-analysis due to non-estimable ORs or CIs. The x-axis is shown on a logarithmic scale.
Supplementary Figure 3. Funnel plots for assessment of publication bias in the meta-analysis. Funnel plots were generated for the comparison of e4*4 vs. e3*3 in Japanese populations with EOAD (A), LOAD (B) and AD (C). Visual inspection did not reveal marked asymmetry; however, interpretation is limited by the relatively small number of included studies. Publication bias was further evaluated using the Begg-Mazumdar, Egger’s, and Harbord-Egger tests (Supplementary Table 4). †, Studies corresponding to Literature IDs L470 (PMID: 7964823) and L376 (PMID: 9701675) were excluded because no e4*4 carriers were present in either the EOAD or control group.

Supplementary Material 2: Supplementary References.

Supplementary Material 3: Supplementary Tables.

## Data Availability

The data supporting the findings of this study were derived from previously published studies and are available in the article and its supplementary materials. The study protocol is publicly available via the Open Science Framework (OSF; https://doi.org/10.17605/OSF.IO/U37QF).

## References

[1] Fortea J, Pegueroles J, Alcolea D, Belbin O, Dols-Icardo O, Vaqué-Alcázar L, et al. APOE4 homozygozity represents a distinct genetic form of Alzheimer’s disease. Nat Med. 2024;30(5):1284–91. 38710950 10.1038/s41591-024-02931-w PMC13310155

[2] Farrer LA, Cupples LA, Haines JL, Hyman B, Kukull WA, Mayeux R, et al. Effects of age, sex, and ethnicity on the association between apolipoprotein E genotype and Alzheimer disease. A meta-analysis. APOE and Alzheimer Disease Meta Analysis Consortium. JAMA. 1997;278(16):1349–56. 9343467

[3] Bertram L, McQueen MB, Mullin K, Blacker D, Tanzi RE. Systematic meta-analyses of Alzheimer disease genetic association studies: the AlzGene database. Nat Genet. 2007;39(1):17–23. 17192785 10.1038/ng1934

[4] Noguchi S, Murakami K, Yamada N. Apolipoprotein E genotype and Alzheimer’s disease. Lancet. 1993;342(8873):737. 8103832 10.1016/0140-6736(93)91728-5

[5] Ueki A, Kawano M, Namba Y, Kawakami M, Ikeda K. A high frequency of apolipoprotein E4 isoprotein in Japanese patients with late-onset nonfamilial Alzheimer’s disease. Neurosci Lett. 1993;163(2):166–8. 8309625 10.1016/0304-3940(93)90373-s

[6] Okuizumi K, Onodera O, Tanaka H, Kobayashi H, Tsuji S, Takahashi H, et al. ApoE-epsilon 4 and early-onset Alzheimer’s. Nat Genet. 1994;7(1):10–1. 8075630 10.1038/ng0594-10b

[7] Yoshizawa T, Yamakawa-Kobayashi K, Komatsuzaki Y, Arinami T, Oguni E, Mizusawa H, et al. Dose-dependent association of apolipoprotein E allele epsilon 4 with late-onset, sporadic Alzheimer’s disease. Ann Neurol. 1994;36(4):656–9. 7944299 10.1002/ana.410360416

[8] Kawamata J, Tanaka S, Shimohama S, Ueda K, Kimura J. Apolipoprotein E polymorphism in Japanese patients with Alzheimer’s disease or vascular dementia. J Neurol Neurosurg Psychiatry. 1994;57(11):1414–6. 7964823 10.1136/jnnp.57.11.1414 PMC1073199

[9] Farrer LA, Cupples LA, van Duijn CM, Kurz A, Zimmer R, Müller U, et al. Apolipoprotein E genotype in patients with Alzheimer’s disease: implications for the risk of dementia among relatives. Ann Neurol. 1995;38(5):797–808. 7486872 10.1002/ana.410380515

[10] Page MJ, McKenzie JE, Bossuyt PM, Boutron I, Hoffmann TC, Mulrow CD, et al. The PRISMA 2020 statement: an updated guideline for reporting systematic reviews. BMJ. 2021;372:n71. 33782057 10.1136/bmj.n71 PMC8005924

[11] Asanomi Y, Shigemizu D, Miyashita A, Mitsumori R, Mori T, Hara N, et al. A rare functional variant of SHARPIN attenuates the inflammatory response and associates with increased risk of late-onset Alzheimer’s disease. Mol Med. 2019;25(1):20. 31216982 10.1186/s10020-019-0090-5 PMC6585023

[12] Miyashita A, Obinata A, Hara N, Mitsumori R, Kaneda D, Hashizume Y, et al. Association of rare APOE missense variants with Alzheimer’s disease in the Japanese population. J Alzheimers Dis. 2025;106(1):363–77. 40397079 10.1177/13872877251340710 PMC12254517

[13] Belloy ME, Andrews SJ, Le Guen Y, Cuccaro M, Farrer LA, Napolioni V, et al. APOE Genotype and Alzheimer Disease Risk Across Age, Sex, and Population Ancestry. JAMA Neurol. 2023;80(12):1284–94. 37930705 10.1001/jamaneurol.2023.3599 PMC10628838

[14] Liu M, Bian C, Zhang J, Wen F. Apolipoprotein E gene polymorphism and Alzheimer’s disease in Chinese population: a meta-analysis. Sci Rep. 2014;4:4383. 24632849 10.1038/srep04383 PMC3955907

[15] Choi KY, Lee JJ, Gunasekaran TI, Kang S, Lee W, Jeong J, et al. APOE Promoter Polymorphism-219T/G is an Effect Modifier of the Influence of APOE ε4 on Alzheimer’s Disease Risk in a Multiracial Sample. J Clin Med. 2019;8(8):1236. 31426376 10.3390/jcm8081236 PMC6723529

