# Supplementary material for: A reappraisal of APOE genetic effects on Alzheimer’s disease risk in the Japanese population: a meta-analysis
Source: Mol Neurodegener. 2026 Jun 24;21:33. doi: 10.1186/s13024-026-00963-z (PMC13292320; doi:10.1186/s13024-026-00963-z)
Supplement: Supplementary file 1 — Supplementary Material 1: Supplementary Figure 1. Flow diagram of study selection and overlap between PubMed and WOS. Flow diagram illustrating study identification, screening, and eligibility assessment for the meta-analysis of APOE genotype effects in Japanese AD. Literature searches were conducted using PubMed and the WOS Core Collection with the query strings shown. A total of 241 records were identified from PubMed and 213 from WOS, and the overlap among records with PMIDs is indicated. After exclusion of non-English articles (n = 14) and studies lacking sufficient APOE genotype data in both case and control groups (n = 200), and after resolving overlapping cohorts (NCGG/JGSCAD and HI-ABCD), 21 eligible studies were finally included in the primary meta-analysis (EOAD, n = 8; LOAD, n = 11; AD without age stratification, n = 10). Full bibliographic details of the 21 included studies are provided in Supplementary References and correspond to the order of the “Literature ID” entries in Supplementary Table 2. Funnel plots for the e4*4 vs. e3*3 comparisons are shown in Supplementary Figure 3, and publication bias statistics for all comparisons are provided in Supplementary Table 4. The two most recent large-scale Japanese studies involving NCGG/JGSCAD subjects were excluded from the primary meta-analysis (Fig. 1A) and retained for reference only, as their substantially larger sample sizes relative to earlier reports could disproportionately influence pooled estimates (Fig. 1B). Supplementary Figure 2. Forest plots of study-specific and pooled ORs (95% CIs) for APOE-e4*4 vs. APOE-e3*3 in Japanese AD. Forest plots showing study-specific ORs with 95% CIs for e4*4 vs. e3*3 (reference) in Japanese EOAD (A, B), LOAD (C, D), and AD (E, F). Fixed-effects model results are prensented in (A), (C), and (E), and random-effects model results in (B), (D), and (F). Squares indicate study-specific point estimates (with size proportional to the study weight in the corresponding meta- [file 13024_2026_963_MOESM1_ESM.pdf]

Miyashita A, *et al*. Supplementary Figure 1

Articles identified:
- PubMed: **241**
- WOS: **213**

PubMed: searched on Saturday, July 5, 2025
"japan*"[Title/Abstract] AND "alzheimer*"[Title/Abstract] AND ("apolipoprotein*"[Title/Abstract] OR "apoe*"[Title/Abstract])

WOS Core Collection: searched on Saturday, March 7, 2026
((TI=(Japan*) OR AB=(Japan*)) AND (TI=(Alzheimer*) OR AB=(Alzheimer*))) AND ((TI=(apolipoprotein*) OR AB=(apolipoprotein*)) OR (TI=(APOE*) OR AB=(APOE*)))

Articles with PMIDs:
- PubMed: **241**
- WOS: **211**

Articles without PMIDs:
- PubMed: **0**
- WOS: **2**

Degree of overlap among the articles with PMIDs

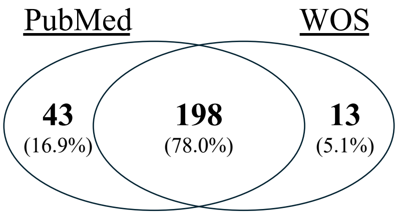

254 articles

Total articles for assessment: **256**

Excluded: **14** (non-English articles [Japanese])

English articles: **242**

Excluded: **1** (two studies included HI-ABCD subjects; the more recent study (Quan *et al.* [2006, PMID: 16233903]) was retained, and the other was excluded.)

241

Excluded: **18** (among studies using NCGG/JGSCAD subjects, the two most recent large-scale studies, Asanomi *et al.* [2019, PMID: 31216982] and Miyashita *et al.* [2025, PMID: 40397079], were retained, and the others were excluded to avoid subject overlap.)

223

Excluded: **200** (studies in which neither the absolute number of subjects nor the frequency data for *APOE* genotypes were available in both the AD and control groups)

Potential articles for meta-analysis: **23**

Excluded: **2** (The two recent studies mentioned above using NCGG/JGSCAD subjects were excluded from the primary meta-analysis to avoid potential bias due to their substantially larger sample sizes compared with earlier reports; these studies were retained and presented separately for reference and comparison in **Figure 1B**)

Eligible studies included in the meta-analysis: **21**
- EOAD: **8**
- LOAD: **11**
- AD: **10**

Miyashita A, *et al*. Supplementary Figure 2

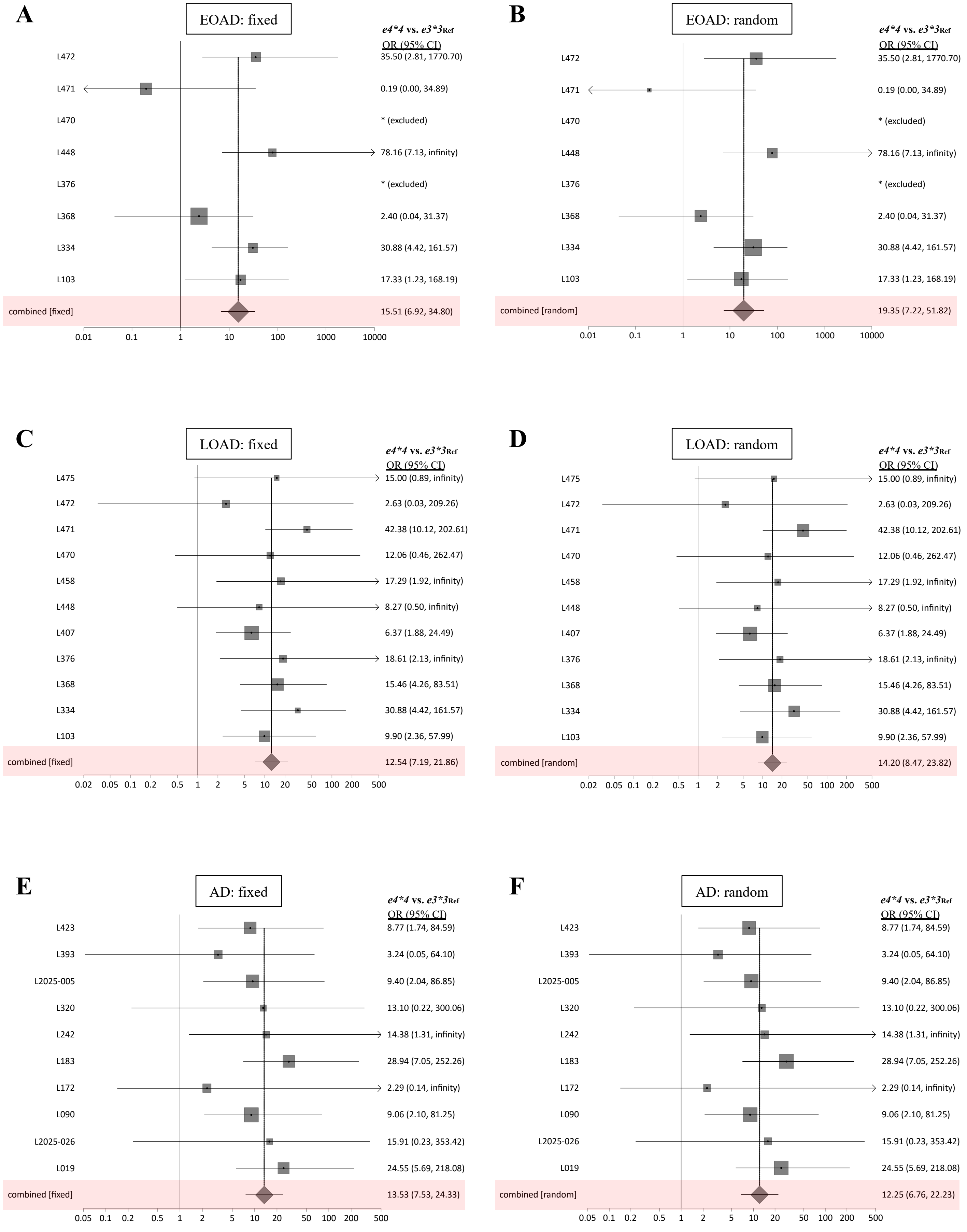

Miyashita A, *et al*. Supplementary Figure 3

A

EOAD: 6 of the 8 studies†

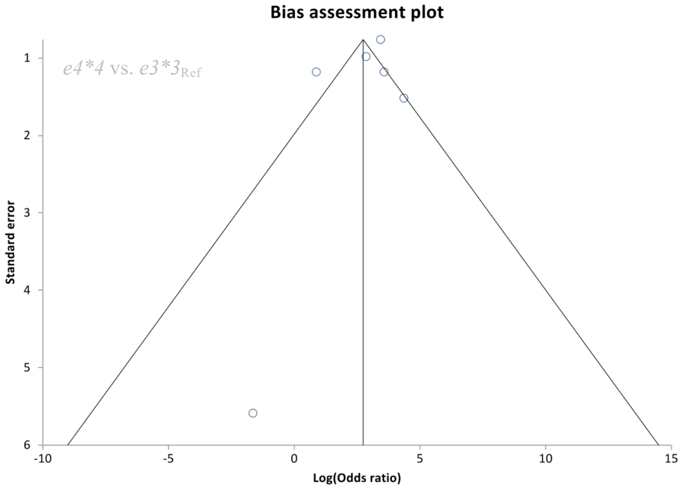

- Begg-Mazumdar test: P = 0.4694
- Egger test: P = 0.5128
- Harbord-Egger test: P = 0.6364

No evidence of publication bias

B

LOAD: 11 studies

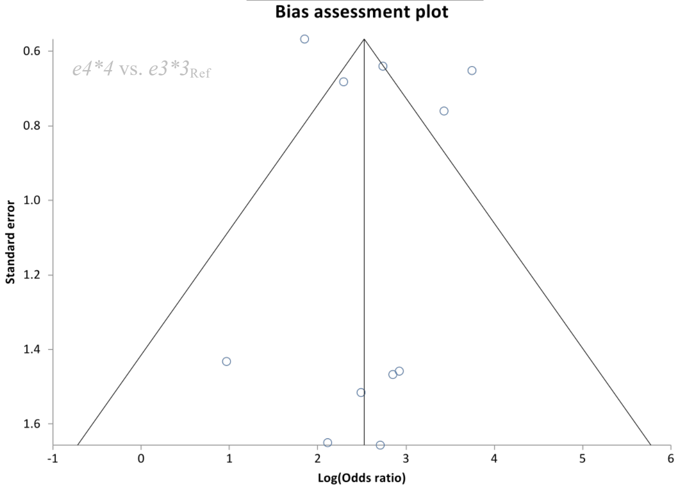

- Begg-Mazumdar test: P = 0.7612
- Egger test: P = 0.7606
- Harbord-Egger test: P = 0.0927

No evidence of publication bias

C

AD: 10 studies

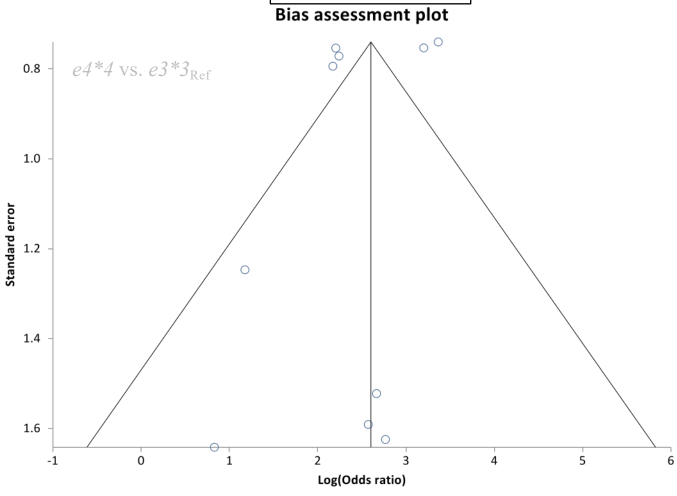

- Begg-Mazumdar test: P = 0.2164
- Egger test: P = 0.2637
- Harbord-Egger test: P = 0.7102

No evidence of publication bias
