# Supplementary material for: A reappraisal of APOE genetic effects on Alzheimer’s disease risk in the Japanese population: a meta-analysis
Source: Mol Neurodegener. 2026 Jun 24;21:33. doi: 10.1186/s13024-026-00963-z (PMC13292320; doi:10.1186/s13024-026-00963-z)
Supplement: Supplementary file 2 — Supplementary Material 2: Supplementary References. [file 13024_2026_963_MOESM2_ESM.pdf]

Supplementary References

Full bibliographic details of all 21 studies included in the meta-analysis are listed below. References are arranged according to the order of the "Literature ID" column in **Supplementary Table 2**.

1. L475: LOAD Ueki A, Kawano M, Namba Y, Kawakami M, Ikeda K. A high frequency of apolipoprotein E4 isoprotein in Japanese patients with late-onset nonfamilial Alzheimer's disease. Neurosci Lett. 1993;163(2):166-8. (PMID: 8309625)
2. L472: EOAD-LOAD Dai XY, Nanko S, Hattori M, Fukuda R, Nagata K, Isse K, Ueki A, Kazamatsuri H. Association of apolipoprotein E4 with sporadic Alzheimer's disease is more pronounced in early onset type. Neurosci Lett. 1994;175(1-2):74-6. (PMID: 7970216)
3. L471: EOAD-LOAD Yoshizawa T, Yamakawa-Kobayashi K, Komatsuzaki Y, Arinami T, Oguni E, Mizusawa H, Shoji S, Hamaguchi H. Dose-dependent association of apolipoprotein E allele epsilon 4 with late-onset, sporadic Alzheimer's disease. Ann Neurol. 1994;36(4):656-9. (PMID: 7944299)
4. L470: EOAD-LOAD Kawamata J, Tanaka S, Shimohama S, Ueda K, Kimura J. Apolipoprotein E polymorphism in Japanese patients with Alzheimer's disease or vascular dementia. J Neurol Neurosurg Psychiatry. 1994;57(11):1414-6. (PMID: 7964823)
5. L458: LOAD Kamino K, Yoshiiwa A, Nishiwaki Y, Nagano K, Yamamoto H, Kobayashi T, Nonomura Y, Yoneda H, Sakai T, Imagawa M, Miki T, Ogihara T. Genetic association study between senile dementia of Alzheimer's type and APOE/C1/C2 gene cluster. Gerontology. 1996;42 Suppl 1:12-9. (PMID: 8804993)
6. L448: EOAD-LOAD Nunomura A, Chiba S, Eto M, Saito M, Makino I, Miyagishi T.

Apolipoprotein E polymorphism and susceptibility to early- and late-onset sporadic Alzheimer’s disease in Hokkaido, the northern part of Japan. Neurosci Lett. 1996;206(1):17-20. (PMID: 8848271)

7. L423: AD Yamagata Z, Asada T, Kinoshita A, Zhang Y, Asaka A. Distribution of apolipoprotein E gene polymorphisms in Japanese patients with Alzheimer’s disease and in Japanese centenarians. Hum Hered. 1997;47(1):22-6. (PMID: 9017975)
8. L407: LOAD Yoshiiwa A, Kamino K, Yamamoto H, Kobayashi T, Imagawa M, Nonomura Y, Yoneda H, Sakai T, Nishiwaki Y, Sato N, Rakugi H, Miki T, Ogihara T. alpha 1-Antichymotrypsin as a risk modifier for late-onset Alzheimer’s disease in Japanese apolipoprotein E epsilon 4 allele carriers. Ann Neurol. 1997;42(1):115-7. (PMID: 9225693)
9. L393: AD Yamanaka H, Kamimura K, Tanahashi H, Takahashi K, Asada T, Tabira T. Genetic risk factors in Japanese Alzheimer’s disease patients: alpha1-ACT, VLDLR, and ApoE. Neurobiol Aging. 1998;19(1 Suppl):S43-6. (PMID: 9562467)
10. L376: EOAD-LOAD Ji Y, Urakami K, Adachi Y, Maeda M, Isoe K, Nakashima K. Apolipoprotein E polymorphism in patients with Alzheimer’s disease, vascular dementia and ischemic cerebrovascular disease. Dement Geriatr Cogn Disord. 1998;9(5):243-5. (PMID: 9701675)
11. L368: EOAD-LOAD Nishimura T, Takeda M, Shinosaki K, Nishikawa T, Nakamura Y, Yoshida Y, Sasaki H, Arai H, Hirai S, Shouji M, Isse K, Tanaka K, Hamamoto M, Yamamoto H, Matsubayashi T, Nakashima K, Urakami K, Adachi Y, Nakamura S, Toji H, Yoshida H. Basic and clinical studies on ApoE gene typing by line probe assay (LiPA) as a biological marker for Alzheimer’s disease and related disorders: multicenter study in Japan. Methods Find Exp Clin Pharmacol. 1998;20(9):793-9. (PMID: 10022033)

12. L334: EOAD-LOAD Nakayama S, Kuzuhara S. Psychiatry Clin Neurosci. Apolipoprotein E phenotypes in healthy normal controls and demented subjects with Alzheimer’s disease and vascular dementia in Mie Prefecture of Japan. 1999;53(6):643-8. (PMID: 10687744)
13. L2025-005: AD Hu J, Miyatake F, Aizu Y, Nakagawa H, Nakamura S, Tamaoka A, Takahash R, Urakami K, Shoji M. Angiotensin-converting enzyme genotype is associated with Alzheimer disease in the Japanese population. Neurosci Lett. 1999;277(1):65-7. (PMID: 10643899)
14. L320: AD Nishiyama M, Kato Y, Hashimoto M, Yukawa S, Omori K. Apolipoprotein E, methylenetetrahydrofolate reductase (MTHFR) mutation and the risk of senile dementia--an epidemiological study using the polymerase chain reaction (PCR) method. J Epidemiol. 2000;10(3):163-72. (PMID: 10860300)
15. L242: AD Yamada M. Risk factors for cerebral amyloid angiopathy in the elderly. Ann N Y Acad Sci. 2002;977:37-44. (PMID: 12480732)
16. L183: AD Quan W, Yasuda M, Hashimoto M, Yamamoto Y, Ishii K, Kazui H, Mori E, Kakigi T, Maeda K. Polymorphism of the regulatory region of the presenilin-2 gene in sporadic Alzheimer’s disease: a case-control study. J Neurol Sci. 2006;240(1-2):71-5. (PMID: 16233903)
17. L172: AD Tamura H, Kawakami H, Kanamoto T, Kato T, Yokoyama T, Sasaki K, Izumi Y, Matsumoto M, Mishima HK. High frequency of open-angle glaucoma in Japanese patients with Alzheimer's disease. J Neurol Sci. 2006;246(1-2):79-83. (PMID: 16564058)
18. L103: EOAD-LOAD Kobayashi S, Tateno M, Park TW, Utsumi K, Sohma H, Ito YM, Kokai Y, Saito T. Apolipoprotein E4 frequencies in a Japanese population with Alzheimer’s disease and dementia with Lewy bodies. PLoS One. 2011;6(4):e18569. (PMID: 21552550)

19. L090: AD Ohnuma T, Nakamura T, Takebayashi Y, Hanzawa R, Kitazawa M, Higashiyama R, Takeda M, Thompson K, Komatsu M, Shimazaki H, Shibata N, Arai H. No Associations Found between PGBD1 and the Age of Onset in Japanese Patients Diagnosed with Sporadic Alzheimer's Disease. Dement Geriatr Cogn Dis Extra. 2012;2(1):496-502. (PMID: 23277782)

20. L2025-026: AD Namiki C, Takita Y, Iwata A, Momose T, Senda M, Okubo Y, Joshi AD, Lu M, Agbulos A, Breault C, Pontecorvo MJ. Imaging characteristics and safety of florbetapir (^{18}F) in Japanese healthy volunteers, patients with mild cognitive impairment and patients with Alzheimer's disease. Ann Nucl Med. 2015;29(7):570-81. (PMID: 25943346)

21. L019: AD Fujishima M, Kawaguchi A, Maikusa N, Kuwano R, Iwatsubo T, Matsuda H; Japanese Alzheimer's Disease Neuroimaging Initiative (ADNI); Japanese Alzheimer's Disease Neuroimaging Initiative (J-ADNI). Sample Size Estimation for Alzheimer's Disease Trials from Japanese ADNI Serial Magnetic Resonance Imaging. J Alzheimers Dis. 2017;56(1):75-88. (PMID: 27911297)
